# Understanding Pitch Perception as a Hierarchical Process with Top-Down Modulation

**DOI:** 10.1371/journal.pcbi.1000301

**Published:** 2009-03-06

**Authors:** Emili Balaguer-Ballester, Nicholas R. Clark, Martin Coath, Katrin Krumbholz, Susan L. Denham

**Affiliations:** 1 Centre for Theoretical and Computational Neuroscience, University of Plymouth, Plymouth, United Kingdom; 2 Computational Neuroscience Group, Central Institute for Mental Health (ZI), Ruprecht-Karls University of Heidelberg, Mannheim, Germany; 3 MRC Institute of Hearing Research, Nottingham, United Kingdom; University College London, United Kingdom

## Abstract

Pitch is one of the most important features of natural sounds, underlying the perception of melody in music and prosody in speech. However, the temporal dynamics of pitch processing are still poorly understood. Previous studies suggest that the auditory system uses a wide range of time scales to integrate pitch-related information and that the effective integration time is both task- and stimulus-dependent. None of the existing models of pitch processing can account for such task- and stimulus-dependent variations in processing time scales. This study presents an idealized neurocomputational model, which provides a unified account of the multiple time scales observed in pitch perception. The model is evaluated using a range of perceptual studies, which have not previously been accounted for by a single model, and new results from a neurophysiological experiment. In contrast to other approaches, the current model contains a hierarchy of integration stages and uses feedback to adapt the effective time scales of processing at each stage in response to changes in the input stimulus. The model has features in common with a *hierarchical generative* process and suggests a key role for efferent connections from central to sub-cortical areas in controlling the temporal dynamics of pitch processing.

## Author Summary

Pitch is one of the most important features of natural sounds. The pitch sensation depends strongly on its temporal context, as happens, for example, in the perception of melody in music and prosody in speech. However, the temporal dynamics of pitch processing are poorly understood. Perceptual studies have shown that there is apparently a wide range of time scales over which pitch-related information is integrated. This multiplicity in perceptual time scales requires a trade-off between temporal resolution and temporal integration, which is not exclusive to pitch perception but applies to auditory perception in general. As far as we are aware, no existing model can account simultaneously for the wide range and stimulus-dependent nature of the perceptual phenomenology. This article presents a neurocomputational model, which explains the temporal resolution–integration trade-off observed in pitch perception in a unified fashion. The main contribution of this work is to propose that top-down, efferent mechanisms are crucial for pitch processing. The model replicates perceptual responses in a wide range of perceptual experiments not simultaneously accounted for by previous approaches. Moreover, it accounts quantitatively for the stimulus-dependent latency of the pitch onset response measured in the auditory cortex.

## Introduction

Modelling the neural processing of pitch is essential for understanding the perceptual phenomenology of music and speech. Pitch, one of the most important features of auditory perception, is usually associated with periodicities in sounds [1]. Hence, a number of models of pitch perception are based upon a temporal analysis of the neural activity evoked by the stimulus [2]–[5]. Most of these models compute a form of short-term autocorrelation of the simulated auditory nerve activity using an exponentially weighted integration time window [6]–[13]. Autocorrelation models have been able to predict the reported pitches of a wide range of complex stimuli. However, choosing an appropriate integration time window has been problematic, and none of the previous models has been able to explain the wide range of time scales encountered in perceptual data in a unified fashion. These data show that, in certain conditions, the auditory system is capable of integrating pitch-related information over time scales of several hundred milliseconds [14]–[22], while at the same time being able to follow changes in pitch or pitch strength with a resolution of only a few milliseconds [14], [15], [21]–[24]. Limits on the temporal resolution of pitch perception have also been explored by determining pitch detection and discrimination performance as a function of frequency modulation rate [25]–[27], the main conclusion being that the auditory system has a limited ability to process rapid variations in pitch.

The trade-off between temporal integration and resolution is not exclusive to pitch perception, but is a general characteristic of auditory temporal processing. For instance, a long integration time of several hundred milliseconds is required to explain the way in which the detectability and perceived loudness of sounds increases with increasing sound duration [28],[29]. In contrast, much shorter integration times are necessary to explain the fact that the auditory system can resolve sound events separated by only a few milliseconds [28]–[30]. Therefore, it appears that the integration time of auditory processing varies with the stimulus and task. Previously it was proposed that integration and resolution reflect processing in separate, parallel streams with different stimulus-independent integration times [28]. More recently, in order to reconcile perceptual data pertaining to temporal integration and resolution tasks, it was suggested that the auditory system makes its decisions based on “multiple looks” at the stimulus [31], using relatively short time windows. However, to our knowledge no model has yet quantitatively explained the stimulus- and task-dependency of integration time constants.

Another major challenge for pitch modelling is to relate perceptual phenomena to neurophysiological data. Functional brain-imaging studies strongly suggest that pitch is processed in a hierarchical manner [32], starting in sub-cortical structures [33] and continuing up through Heschl's Gyrus on to the *planum polare* and *planum temporale* [34]–[36]. Within this processing hierarchy, there is an increasing dispersion in response latency, with lower pitches eliciting longer response latencies than higher pitches [37]. This suggests that the time window over which the auditory system integrates pitch-related information depends on the pitch itself. However, no attempt has yet been made to explain this latency dispersion.

In this study, we present a unified account of the multiple time scales involved in pitch processing. We suggest that top-down modulation within a hierarchical processing structure is important for explaining the stimulus-dependency of the effective integration time for extracting pitch information. A highly idealized model, formulated in terms of interacting neural ensembles, is presented. The model represents a natural extension of previous autocorrelation models of pitch in a form resembling a *hierarchical generative* process [38],[39], in which higher (e.g., cortical) levels modulate the responses in lower (e.g., sub-cortical) levels via feedback connections. Without modification, the model can account not only for a wide range of perceptual data, but also for novel neurophysiological data on pitch processing.

## Methods

**Figure 1:**
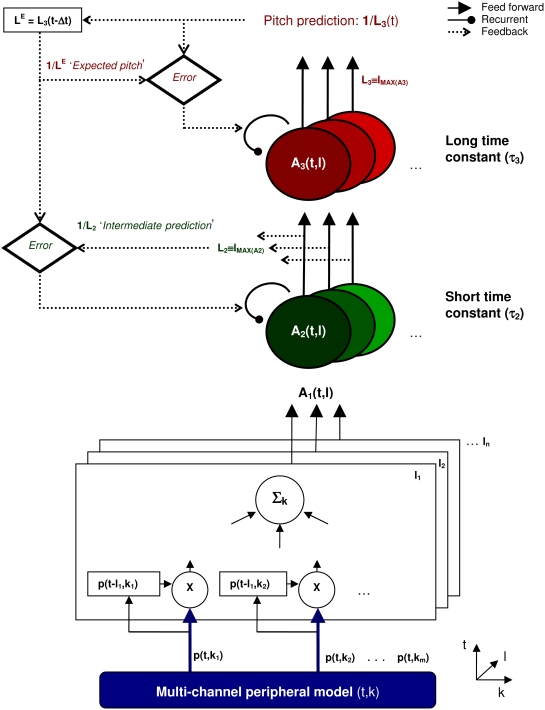
Schematic outline of the model. The model consists of: 1) a simulation of auditory nerve spiking probabilities, *p*(*t,k*) (blue), in response to a sound for each cochlear frequency channel, *k*; 2) a cross-product of the auditory nerve activity with a time-delayed version of itself for a range of different time lags, *l* (in the diagram, processing relating to different lags is represented by stacked boxes); 3) two integration stages, *A_2_ and A_3_*, shown by green and red ellipses, which represent highly idealized models of collective neuronal responses using a shorter (τ_2_) and a longer (τ_3_) time constant, respectively. *L_2_*(*t*) is the lag yielding the maximum response at the second processing stage, *A_2_*(*t,l*); its inverse, *1/L_2_*(*t*) represents an intermediate pitch prediction of the model. Similarly, *1/L_3_*(*t*) represents the ultimate pitch estimate predicted by the model. When the pitch estimate changes over time, a mismatch between the previous pitch estimate at level 3 (labelled “expected pitch” or 1/*L^E^*) and the current prediction at the first integration stage, *1/L_2_*, feeds back to modulate the recurrent processes (curved lines) at both integration stages. See text for details.

The model consists of a feed-forward process, as well as a feedback process, which modifies the parameters of feed-forward processing. Both components are explained in detail below and schematic diagram of the model is shown in Figure 1.

### Feed-Forward Processing

The role of the feed-forward process (solid lines in Figure 1) is to predict the pitch of the incoming stimulus. The perceived pitch of periodic sounds corresponds approximately to the reciprocal of the repetition period of the sound waveform. This is why temporal models of pitch perception, such as autocorrelation models, usually analyze the periodicities of the signal within the auditory-nerve channels, and then use these periodicities to derive a pitch estimate by computing the reciprocal of the periodicity that is most prevalent across frequency channels [2].

The cochlea in the inner ear acts as a frequency analyzer, in that different sound frequencies activate different places along the cochlea, which are in turn innervated by different auditory nerve fibres [1]. Thus, the cochlea can be modelled as a bank of band-pass filters. In the current model, each cochlear filter was implemented as a *dual resonant nonlinear gammatone filter*, which accounts for the sound level-dependent non-linear properties of cochlear processing [40]. The filter output was then passed through a hair cell transduction model [41] to simulate the conversion of the mechanical cochlear response into auditory-nerve spiking activity. The model was implemented using DSAM (Development System for Auditory Modelling http://www.pdn.cam.ac.uk/groups/dsam/). It contained a total of 30 frequency channels with centre frequencies ranging from 100 to 10000 Hz on a logarithmic scale.

The hair cell transduction model generates auditory-nerve spike probabilities, *p*(*t,k*), as a function of time, *t*, in each frequency channel, *k*. The first processing stage (open boxes in Figure 1) computes the joint probability that a given auditory nerve fibre produces two spikes, one at time *t* and another at *t-l*, where *l* is a time delay or lag [10]. These joint probabilities are generated by computing the cross-product of the auditory-nerve firing probability, p(t,*k*), with time-delayed versions of itself for a range of time delays. The cross-products are then summed across all frequency channels, *k*, to generate the output of the first stage of the model *A_1_*(*t,l*):
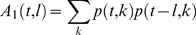
(1)


The activity at the second processing stage, *A_2_*(*t,l*) (green circles in Figure 1), is computed as a leaky integration, (i.e., a low-pass filter using an exponentially decaying function [42]) of the input activity, *A_1_*(*t,l*), using relatively short time constants, τ_2_. It may therefore be assumed to represent sub-thalamic neural populations [43]–[46]. The time constants at the second stage are lag-dependent (τ_2_ = τ_2_(*l*)), as suggested by recent psychoacoustic studies [23],[37]. However, for clarity, the lag dependency will not be explicitly stated in the following equations. In the third stage, *A_3_*(*t,l*) (red circles in Figure 1), the output of the second stage is integrated over a longer time scale, τ*_3_*, as suggested by neuroimaging studies of pitch in the cortex [37],[47]. This stage is assumed to be located more centrally. Both integration stages can be simply described as time-varying exponential averages,
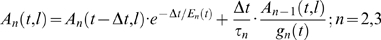
(2)


In equation (2), Δ*t* is the time step of the integration and *E_n_*(*t*) is the instantaneous exponential decay rate of the response at each integration stage (*E_n_*(*t*)≤τ*_n_*), which will henceforth be referred to as the *effective integration window*. Establishing an appropriate time constant is as has been mentioned one of the major difficulties in formulating a general model of pitch perception. Hence, the value of *E_n_*(*t*) in the model proposed here is not constant but is controlled by changes in the properties of the stimulus. The control of *E_n_*(*t*) will be explained below.

The factors *g_n_*(*t*) normalize the input to each stage by the corresponding integration window (*g_2_*≡*1*; *g_3_*(*t*)* = E_2_*(*t*)*/*τ*_2_*).

At each time step *A_n_*(*t,l*) will have a maximum at some value of *l* which we will write as *L_n_*. The inverse of this lag for the output of stage 2, *1/L_2_*(*t*), represents the intermediate pitch prediction of the model (see Figure 1). Similarly, the inverse of the lag corresponding to the maximum response in stage 3, *1/L_3_*(*t*) is the final pitch prediction. For convenience, we refer to the final pitch prediction from the preceding time step *1/L_3_*(*t*-Δt) as the pitch *expectation*, *1/L^E^*. In all simulations presented in the current study, we used 200 lags, with reciprocals logarithmically distributed, representing pitches between 50 to 2000 Hz [48].

**Figure 2:**
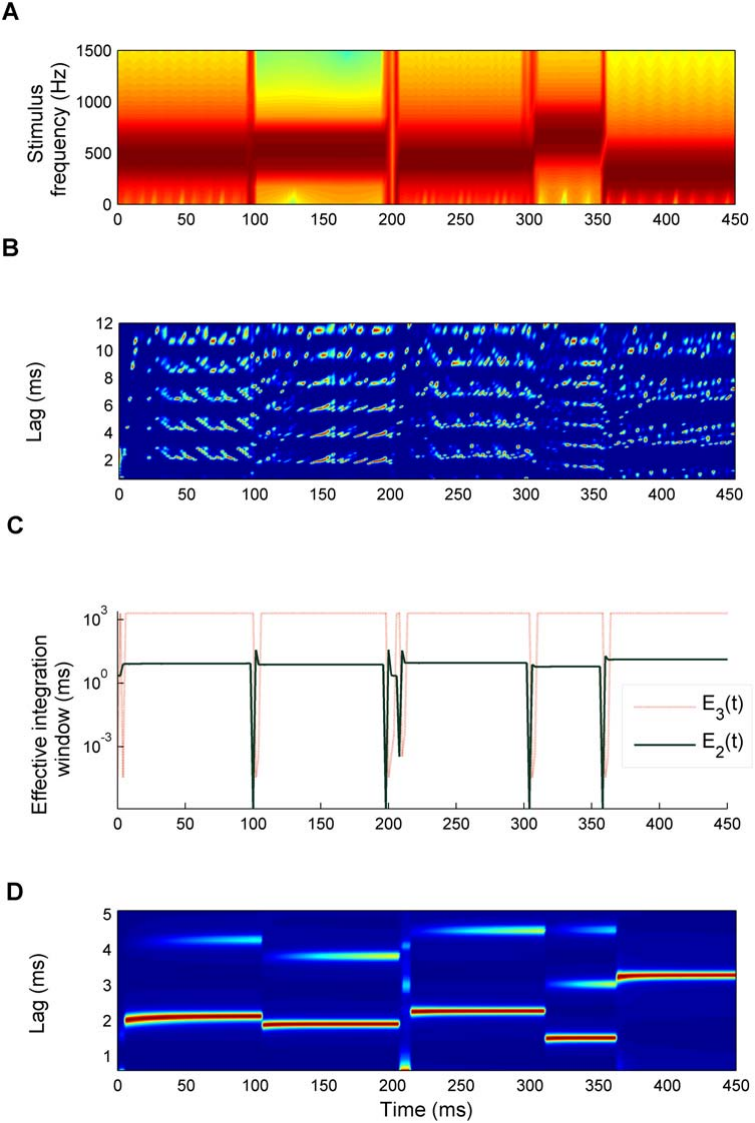
Example of the model output in response to an arbitrary sequence of pure tones with random frequencies and durations. (A) Spectrogram of the stimulus as a function of time. (B) Response of the second processing stage, *A_1_*(*t,l*), plotted as a function of time, *t* (abscissa), and time lag, *l* (ordinate). (C) Effective integration window of the second and third processing stages, *A_2_*(*t,l*) (*E_2_*(*t*), green solid line) and *A_3_*(*t,l*) (*E_3_*(*t*), red dotted line). *E_2_*(*t*) represents the integration time at the lag corresponding to the maximum response in the second stage. (D) Response of the third processing stage, *A_3_*(*t,l*). *A_3_*(*t,l*) was normalized to a maximum response of unity and exponentially enhanced after each time step for illustrative purposes. The colours in plots (B) and (D) represent the activation strength as a percentage of the maximum response at that time (blue: low response or 0%; red: maximal response or 100%). Thus, the lag channel corresponding to the current pitch estimate appears red.

As an example, Figure 2 shows the model response to a sequence of pure tones (Figure 2A) with random frequencies and durations. Figure 2B shows the first stage of the model *A_1_*(*t,l*) and Figure 2C the effective integration windows. Figure 2D shows the final model output; the red colour highlights the lag-channels with strong responses. The lag of the channel with the maximum response at a given time corresponds to the reciprocal of the pitch predicted by the model. Note that the response *A_3_*(*t,l*) in Figure 2D was normalized to a maximum of unity after each time step and mapped exponentially onto the colour scale to make the plot clearer. However, this transformation is monotonic and thus does not affect the model predictions.

**Figure 3:**
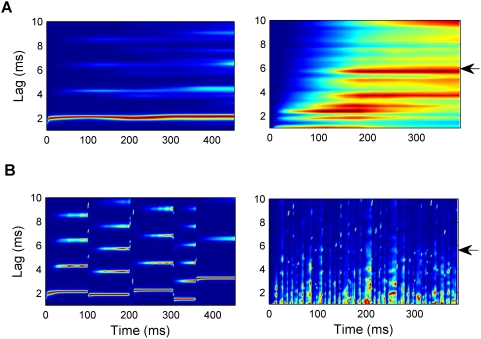
Responses of autocorrelation models with fixed time constants. (A) Response of the cascade autocorrelation model [13]; left plot: to the sequence of random tones shown in Figure 2A, and right plot: to the alternating click train shown in Figure 8A. (B) Response of short-term integration stage of the cascaded model (corresponding to the second stage of the current model, *A_2_*(*t,l*), when the feedback modulation of the integration times, equation 8, is switched off); see text for further explanation. As in panel A, the left panel shows the response to the tone sequence and the right panel shows the response to the click train (arrows mark the reported pitches). Different colours show activation strength as a percentage of the maximum response, as in Figure 2.

The necessity for stimulus-driven modulation of the effective integration time, *E_n_*(*t*), becomes clear from a consideration of existing autocorrelation models. If *E_2_*(*t*) were constant over time, i.e., *E_2_*(*t*) ≡ τ*_2_* , then *A_2_*(*t,l*) would correspond to the *summary autocorrelation function* (SACF) proposed by Meddis and colleagues [6],[7]. If, in addition, *E_3_*(*t*) ≡ τ*_3_* then *A_3_*(*t,l*) would represent an additional leaky integrator with a longer time constant. This is equivalent to the *cascade autocorrelation model* proposed by Balaguer-Ballester et al. [13]. The right panel in Figure 3A illustrates the success of the purely feed-forward model in response to a click train stimulus with alternating inter-click intervals [49],[50]. The arrow indicates the average pitch reported by listeners. The pitch of such alternating click train stimuli has been difficult to predict with autocorrelation models consisting of only one integration stage with a short time constant (see right panel in Figure 3B).

However, the longer time scale used in the second stage of the cascade autocorrelation model prevents the detection of rapid pitch changes such as in the sequence of pure tones shown in Figure 2. The left panel in Figure 3A clearly shows that the cascade autocorrelation model fails to distinguish the pitches of individual tones in the tone sequence used in Figure 2, while the left panel in Figure 3B shows that the SACF model does so fairly well. Therefore, stimulus-dependent changes in the effective integration windows are required.

### Parallels with Population Models

Autocorrelation is usually considered to be a simplified phenomenological model of pitch perception, which is not straightforward to implement in a biologically plausible way [8],[43]. This is also the case for the proposed model. Nevertheless, an alternative, more formal way to express the second and third model stages (equation 2) is shown in equation (3), below. This is equivalent to an expression for the response of a neural population which integrates activity from the previous stage [42]:
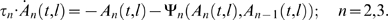
(3)


The dot indicates a partial temporal derivative and τ*_n_* is defined as the processing time constant of an idealized homogeneous population of neurons at stage *n*. The “activation” functions,Ψ*_n_*, in equation (3), which typically use a fixed sigmoid function in standard models of neural assembles [51], are in the model proposed here time-dependent multiplicative gains:
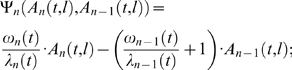
(4)where ω*_1_/*λ*_1_*≡*0*; and ω*_n,_* λ*_n_* are defined in the next section.

Substituting equation (4) into equation (3) and integrating, allows us to obtain the effective integration windows, *E_n_*(*t*), used in equation (2):
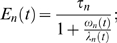
(5)


### Detecting Changes in the Stimulus

In contrast with the feed-forward model, the goal of the feedback processing (dotted lines in Figure 1) is to detect unexpected changes in the input stimulus, such as the offset of a tone in a sequence, and to modulate the integration times involved in the feed-forward processing when such changes occur.

In the case where the stimulus is constant the pitch predictions at successive time steps will not differ. However, if the stimulus changes then the height of the peak corresponding to the current pitch prediction *1/L_n_*(*t*) will change from one time step to the next. A mismatch between the pitch predictions at each level and the pitch expectation therefore indicates a change in the input stimulus.

A stimulus change typically requires a fast system response, so that information occurring around the time of the change can be updated quickly; this corresponds to using small *E_n_*(*t*) values. Thus, during periods when there is a significant discrepancy between the current and expected pitch estimates, the effective integration time windows at both integration stages should become very short, so that the “memory” component of the model response is reduced to near zero and essentially reset. Similar rapid changes of activity in response to variations in the input have been previously reported in neural ensemble models [51],[52].

Figure 2C illustrates the dynamics of *E_2_*(*t*) (solid green line) and *E_3_*(*t*) (dotted red line) in response to a random tone sequence, the spectrogram of which is shown in Figure 2A. After the end of each tone, both time constants, *E_2_* and *E_3_*, decrease for a brief period of time and then recover back to their maximum values (*E_n_*(*t*)≈τ*_n_*) when the next tone begins. As *E_2_* is lag-dependent, the values plotted in Figure 2C represent the integration time constant at the lag, *L_2_*(*t*), corresponding to the current maximum of *A_2_*(*t,l*). The small overshoots after the initial dips in *E_2_* reflect transient variations in *L_2_* before a new stable prediction is achieved.

The effective integration windows, *E_n_*(*t*), can vary over a large range of values, far exceeding the range of plausible neural time constants. However, it should be noted that the neural processing time constants used in the model, *τ_n_* (see equation 3), only take on biologically plausible values (shown in Table 1). The effective integration windows, derived from the activation functions (equation 5), do not represent neural processing time constants. This aspect will be further addressed in the Discussion section.

**Table 1:** Model parameters used in the simulations.

Parameter	θ*_2_*	θ*_3_*	τ*_2_*(*l*) (ms)	τ*_3_* (ms)	η*_2_* (kHz)	η*_3_* (kHz)	μ*_2_* (kHz)	μ*_3_* (kHz)
Value	0.04	0.07	2–80	2000	3.55	1.15	0.18	1.15

Thresholds θ*_n_* are dimensionless. Sampling frequency of the sounds (1*/Δt*) was 176 kHz; integration period in level three was 2 ms. τ_n_>λ_n_(t)>10^−9^ ms.

During the steady-state portions of each tone, the model essentially behaves like the cascade autocorrelation model [13]. The feedback mechanism simply allows the model to adapt quickly to changes in the stimulus.

A natural measure of the mismatch between pitch expectations and pitch predictions is the *relative error gradient* of the maximum response in *A_n_*(*t,L_n_*),
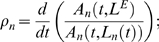
(6)where the expected lag, *L^E^*, is fixed in the temporal derivative; and *L_n_*(*t*) is the lag corresponding to the maximum response at each time step as defined earlier.

The gradient at stage three in the model, 
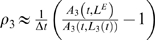
 is an “error” measure: if there is mismatch between the expected pitch estimate and the current prediction, i.e., *L^E^ ≠ L_3_*(*t*), then ρ*_3_<0*. Similarly, at the second stage, ρ*_2_<0* represents a mismatch, or error, between the expected pitch and the current intermediate prediction at stage two, 1/*L_2_*(*t*).

### Feedback Modulation

The goal of the feedback modulation triggered by changes in the stimulus is to adjust the effective time constants *E_n_*(*t*). The error gradients ρ*_n_* give us a measure of stimulus change therefore, when ρ*_n_* is negative *enough* (compared to a threshold value θ*_n_*) there is a discrepancy between the pitch prediction and the pitch expectation which requires that the time constants be adjusted. This is achieved by temporarily activating the recurrent term in equation 4, i.e., by defining
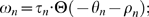
(7)where Θ(*x*) is the Heaviside function (equal to unity if *x>0* and zero otherwise) and θ*_n_* are small positive thresholds for the error terms, ρ*_n_*. For example, during the gaps between tones in a sequence of tones, ρ*_n_<*−θ*_n_* and the gains ω*_n_*(*t*)*/*λ*_n_*(*t*) temporarily become nonzero, thereby modulating the effective temporal integration windows, *E_n_*(*t*).

This approach leads to a problem with the model as described so far in that the response to stimuli where there is a continuous discrepancy between expectations and predictions, very short effective time windows (*E_n_*(*t*)≪τ*_n_*) produce oscillatory responses which do not correspond to the stable pitch perceived by listeners (see, for example, Figure 3B, right panel). The dynamics of the ‘adaptation’ variable, λ*_n_*(*t*), defined in equation 8 below, serve to modulate uncontrolled corrections to the effective integration windows.

Initially the value of λ*_n_*(*t*) is small (λ*_n_*(*0*)≪τ*_n_*) so that when change is first detected *E_n_*(*t*) also becomes small (equation 5). However, in situations where there is a continuous mismatch between the predicted and the expected pitch, λ*_n_*(*t*) grows and *E_n_*(*t*) recovers to a value closer to τ_n_.

Then, when there is no longer any discrepancy between expectation and prediction, λ*_n_*(*t*) recovers to a small value again but without affecting *E_n_*(*t*) because, in the absence of a mismatch, ω_n_ *= 0*. Therefore, the dynamics of λ are described in general by:
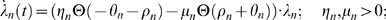
(8)


Where η and μ are the constants that control the rate of increase in λ during periods of mismatch and the rate of decay in λ during periods where no mismatch occurs.

**Figure 4:**
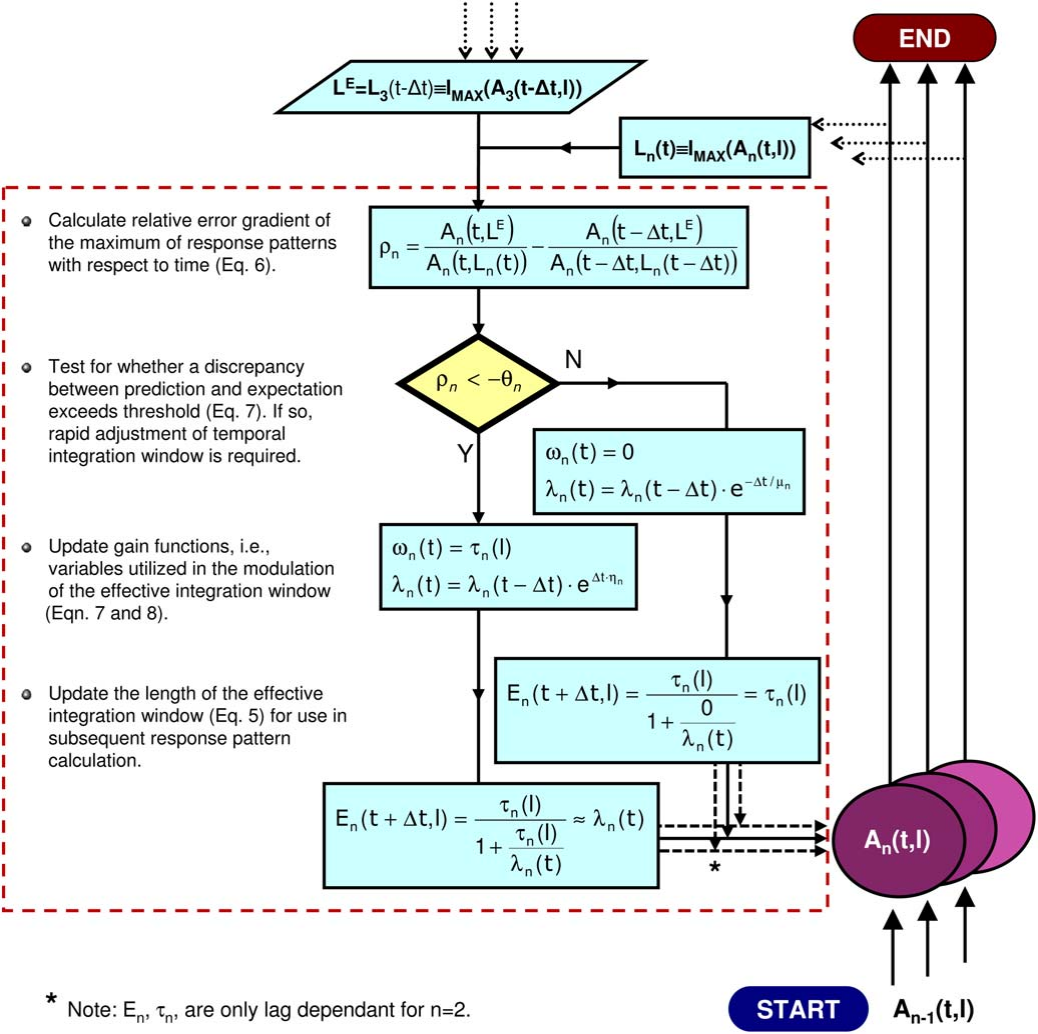
Diagrammatic representation of the computations involved in the recurrent processes of *A_n_*(*t,l*) in flowchart form.

Figures 2C and 8B illustrate two opposite instances of the effect of this top-down processing. In response to a sequence of tones, the effective integration windows shorten precisely at the tone offsets before returning to their maximum values, τ*_n_*, during the tones (Figure 2C). In response to a click train with alternating inter-click intervals (Figure 8B), the window length settles to a maximum value after a longer period of transient fluctuations. Figure 4 illustrates the discrete processing steps of the model in the form of a flowchart. Table 1 gives the set of parameter values used in the simulations. Further neurobiological justifications for the model are presented in the Discussion. A Matlab-based software implementation of the model is freely available from the first author.

## Results

The model was evaluated using a representative set of psychophysical experiments, which illustrate the different time scales of temporal integration and resolution in pitch perception. A further experiment was conducted specifically for this study. Finally, the last evaluation shows that the proposed model can also replicate neurophysiological data.

### Global Pitch of Non-Simultaneous Tones

**Figure 5:**
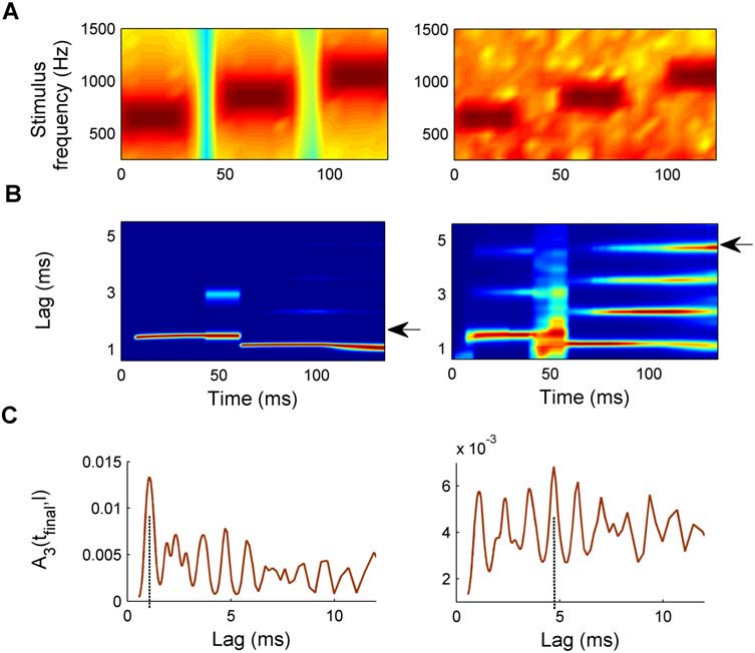
Response of the model to stimuli used in the Hall and Peters' experiment [14]. (A) Spectrogram of a rapid sequence of three 40-ms tones presented in quiet (left panel) and after the addition of white noise (right panel). (B) Response of the third stage of the model, *A_3_*(*t,l*), for the stimulus in quiet (left panel) and in noise (right panel). In the noise condition, the response represents the average over three different random realizations of the noise background. Different colours represent activation strength as a percentage of the maximum response as in previous figures. Arrows indicate the lowest pitch reported by listeners in each condition. (C) Snapshot of *A_3_*(*t,l*) at the end of the stimulus (*t_final_*) in quiet (left panel) and in noise (right panel). Vertical dashed lines correspond to the final predicted pitch.

Hall and Peters' experiment highlighted an unsolved problem concerning the balance between synthetic and analytic listening in response to a sequence of pure tones [14],[15]. The stimuli of the pioneering Hall and Peters' study [14] consisted of three tones played sequentially either in quiet (Figure 5A, left panel) or against a background of white noise (Figure 5A, right panel). Each tone lasted 40 ms and was separated from the following tone by a gap of 10 ms. Tone frequencies were 650, 850 and 1050 Hz (similar results were obtained with a harmonic sequence). The overall level of the noise was about 15 dB above the level of the tones. The individual tones in the sequence were perceived in both conditions.

In the experiment, listeners were instructed to match the *lowest* pitch that they perceived, and in the quiet condition, this was the first of the tones (650 Hz). However, in the noise condition, the non-simultaneous tones combine to create a lower global pitch of about 213 Hz, which is not perceived in the quiet condition. Recently, it was shown that the cascade autocorrelation model, which used two fixed integration stages, could account for the perception of the global pitch in the noise condition when the time constant of the second stage was long enough [13]. However, the same, long, integration stage could not be used to simultaneously predict the perception of the individual tones in quiet.

Figure 5B shows the responses *A_3_*(*t,l*) over time. As in Figure 2, the responses after each time step have been normalized for visualization purposes (however, it should be noted that their real magnitudes, which are close to zero during the silent gaps, are not evident in the figure). The maximum of *A_3_*(*t,l*) correctly predicts the pitches perceived in quiet, which correspond approximately to the frequencies of the individual tones at each moment in time (left plot). Thus, the peak in the profile of the final response at the end of the stimulus correctly reflects the period of the last tone of the sequence at 0.95 ms, and the lowest reported pitch corresponds to the first tone in the sequence (horizontal arrow in Figure 5B).

However, when background noise is present (Figure 5B, right plot), a global pitch gradually emerges (horizontal arrow in the right plot), and the peak in the final response occurs at the reciprocal of the perceived pitch of 213 Hz (4.7 ms, right panel of Figure 5C). The above results match precisely the listeners' responses in this study [14].

**Figure 6:**
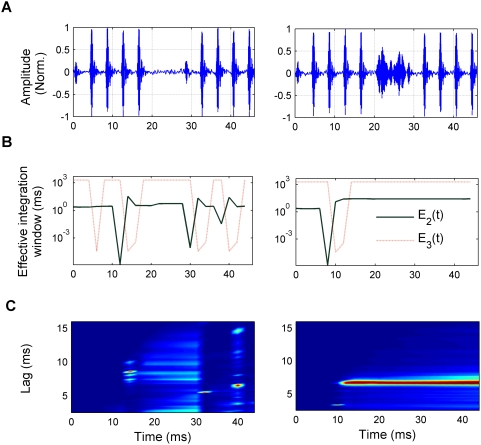
Response of the model to stimuli used in the Plack and White experiment [17]. (A) Stimulus waveform of a rapid sequence of two 20-ms complex tones (harmonics of 250 Hz) separated by a 8-ms silent gap (left panel) or a noise of similar root mean square level as the complex tones (right panel); after band pass filtering (5500–7500 Hz) and the addition of a white noise background. (B) Effective integration times, *E_2_*(*t*) (green solid line) and *E_3_*(*t*) (red dotted line) at the second and third stages of the model. (C) Response of the third stage of the model, *A_3_*(*t,l*), for the silent-gap condition (left panel) and noise-burst condition (right panel). Different colours represent activation strength as a percentage of the maximum response as in previous figures.

Many other studies have explored more explicitly the characteristics of temporal integration in pitch perception. Earlier findings showed that the accuracy of pitch discrimination increases with stimulus duration [1],[19], depends on the resolvability of the harmonics [20], and on the sudden onsets and offsets of overlapping tones [21],[22]. In Figure 6, another example of the model's ability to simulate the integration of pitch information across noise-filled gaps is presented [17],[18]. Figure 6A shows a sequence of two unresolved complex tones of 20-ms duration, containing 100 harmonics of a 250-Hz base frequency, high-pass filtered from 5500 to 7500 Hz. After the first of the tones, there was either a short silent gap (silent-gap condition) or a noise-filled gap, having a similar mean level to the harmonic complex (noise-burst condition). Background noise was added to mask distortion products. In their study, Plack and White reported that subjects perceived pitch continuity through the gap in the noise-burst condition, but not in the silent-gap condition [17]. The normalized model output *A_3_*(*t,l*) (Figure 6C) is qualitatively consistent with a continuous pitch sensation in the noise-burst condition (right panel), which does not occur in the silent-gap condition (left panel).

Conditions under which pitch encoding is affected by the presence of other sounds have been also studied using non-simultaneous stimuli such as temporal “fringes” (consisting of complex tones played immediately before and after a “target” tone) [16],[53],[54]; and by mistuning delayed harmonics of the complex [12], [55]–[57]. The model described here also accounts for the “reset” of pitch information occurring for large frequency differences between fringe and target tones [53] (data not shown).

### Temporal Resolution for Pitch Information

The previous section shows the model's ability to generate stimulus-dependent changes in the effective time scale of temporal integration for extracting pitch information. This raises the question of whether the ability of the model to adjust the effective integration windows could also account for the temporal resolution of the auditory system. While there is substantial evidence for temporal integration in pitch perception, temporal resolution in pitch perception is perhaps still poorly understood. Therefore, we conducted a psychoacoustic experiment specifically to investigate the temporal resolution of pitch information. It should be stressed that this experiment was conducted independently of the model development and was subsequently used to test the model's *predictions*.

#### Psychoacoustic study

Thresholds were measured in two experimental conditions, designed to assess the temporal resolution of the auditory system to changes in pitch strength. In both conditions, a stimulus referred to as rippled noise (RN) was used. RN is generated by delaying a random noise by a delay, *d*, and adding the delayed copy back to the original noise [58]. This delay-and-add process creates a degree of serial correlation in the noise stimulus. When the delay is between about 1 and 30 ms, this correlation gives rise to the perception of a buzzy tone with a pitch corresponding to the reciprocal of the delay, 1/*d*. The serial correlation, and thus the pitch, of RN can be switched on and off by replacing portions of the delayed noise by an uncorrelated noise of the same intensity. In the first condition, referred to as the gap condition, serial correlation was switched off for a single, brief period around the temporal centre of the stimulus, and the shortest detectable gap in correlation, referred to as the pitch-gap detection threshold, was measured.

**Figure 7:**
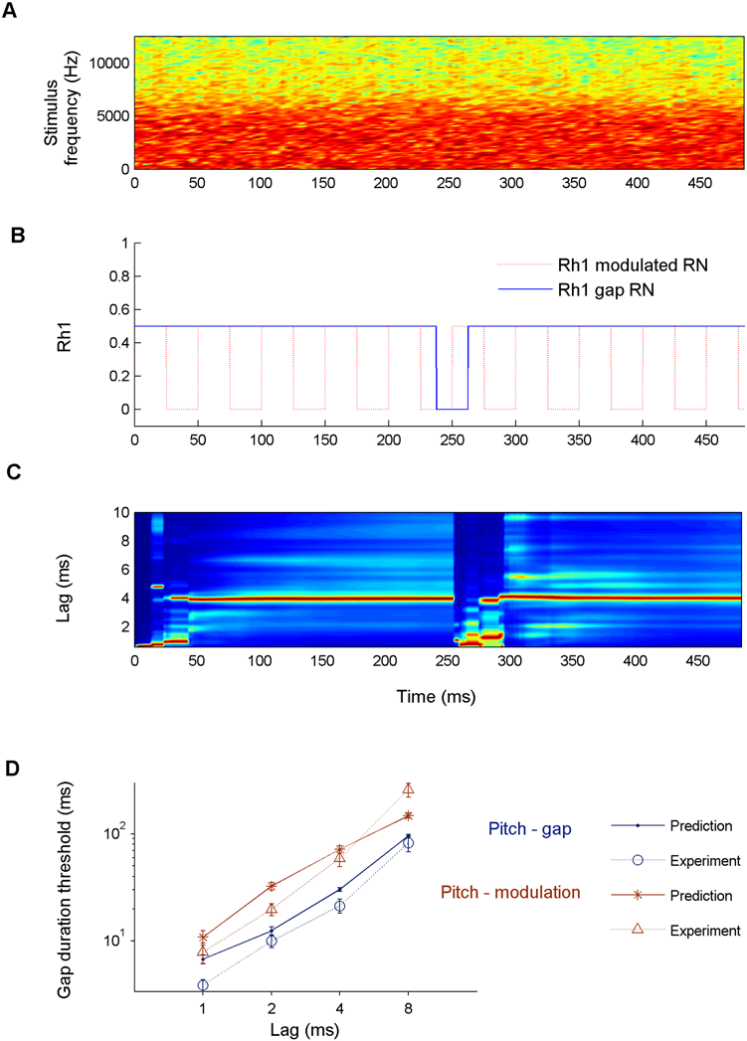
Comparison between human and model pitch-gap and pitch-modulation thresholds in a task specifically designed for assessing temporal resolution in pitch perception (see text for details). (A) Spectrogram for a rippled noise (RN) with a 4-ms delay, which contains a 25-ms gap in serial correlation around the centre of the stimulus, not visible in the figure. (B) First peak height of the running autocorrelation as a function of time (Rh1[t]) for both the modulated (red) and gap (blue) RN stimuli; averaged over 10^5^ stimulus realizations. (C) *A_3_*(*t,l*), for the stimulus shown in panel A, normalized and displayed as in previous figures. (D) Average detection thresholds and standard errors for the pitch-gap (blue circles) and pitch-modulation conditions (red triangles). The corresponding model predictions are shown in the same colours (dots and stars).

In the second condition, referred to as the modulation condition, correlation was switched on and off periodically according to a square-wave function with a 50% duty cycle (i.e., the proportion of time for which correlation was high). In this case, the pitch-modulation detection threshold was measured. This threshold is the fastest rate at which the modulation in correlation was just detectable. Both the pitch-gap and pitch-modulation detection thresholds were measured for four different values of the RN delay, *d* (1, 2, 4, 8, 12 and 16 ms). Figure 7A shows an example of a RN stimulus for *d* = 4 ms in which the gap in correlation is 25 ms. Note that the gap is not visible in the spectrogram. Figure 7B shows the first peak height of the average running autocorrelation as a function of time (Rh1[t]) for both the modulated (red) and gap (blue) RN stimuli of the same delay and gap sizes. Note that panels A and C in Figure 7 refer to the gap stimulus alone.

Thresholds were obtained using a two-interval, two-alternative forced-choice (2I2AFC) adaptive procedure using a 3-down 1-up rule [59]. Stimuli had a duration of 1 s; they were low pass filtered at 5 kHz (24 dB/oct.) and presented at a level of 65 dB SPL (decibel sound pressure level). A minimum of three threshold estimates were obtained for each condition for each of five participants. Figure 7D shows the average gap (blue circles) and modulation detection thresholds (red triangles) with standard errors as a function of the RN delay, *d*, (for computational costs in the model simulations, we only show the results up to *d = *8 ms). Stimuli were generated digitally and converted into analogue signals with a 24-bit amplitude resolution and a sampling rate of 25 kHz using TDT System 3 (Tucker-Davies Technology, Alachua, FL, USA) and Matlab (The Mathworks, Natick, MA, USA). They were amplified (TDT HB7) and presented over headphones (K240 DF, AKG, Vienna, Austria) to the participant, who was seated in a double walled sound attenuating room.

#### Model predictions

Figure 7C illustrates *A_3_*(*t,l*) (normalized at each time step as in previous figures) in response to the stimulus shown in Figure 7A as a function of autocorrelation lag. While the 25-ms uncorrelated noise portion is not visually appreciable in the stimulus spectrogram (Figure 7A), the gap (Figure 7B, blue line) is audible. Consistent with perception, the predicted pitch (red highlight in Figure 7C) shows a discontinuity around the position of the gap in the stimulus.

The blue dots and red stars in Figure 7D show the model predictions, averaged over 100 stimulus realizations for each condition. When the gap or modulation led to discontinuities in the pitch predicted by the model (as shown in Figure 7C), the gap or modulation was considered to be “detectable” by the model. The criteria for detecting a discontinuity were as follows: the predicted pitch around the midpoint of the stimulus duration changed by at least a semitone, and the duration of the detected discontinuity was greater than 4 ms.

Using the above criteria, the predicted thresholds qualitatively match the listeners' mean detection thresholds in both tasks (gap and modulation detection; the modelled and measured thresholds were statistically indistinguishable for all but two of the delays tested). Importantly, it can be seen that, for each RN delay, the model predicted the thresholds for the modulation detection task to be significantly greater than that for the gap detection task (solid lines), as was indeed the case in the data (dotted lines); i.e., the perception of the discontinuity is more difficult when modulations occur periodically rather than only once. This result is somewhat counterintuitive, because the modulation condition contains similar information to the gap condition (see Figure 7B), but repeated over time. It would be difficult to explain this using a conventional autocorrelation model with a single integration time constant. In such a model, the presence of a short discontinuity could only be detected by using a short time constant. Therefore, *A_3_*(*t,l*) would only reflect the recent stimulus history and not the influence of previous modulation cycles. However, in the model reported here, the stimulus-dependency of the effective time constants allows the model to capture both the short term disruptions and the longer term contextual influence and thereby the perceptual differences in pitch-gap and pitch-modulation conditions (Figure 7D).

### Pitch of Click Train Stimuli

Figure 2B showed that the model uses very short integration times for pitch information when a change in pitch occurs. However, it is possible to construct a class of stimuli, in which the periodicities change continually over very short time scales but which nevertheless elicit a single pitch [49],[50], suggesting that pitch information is integrated across these rapid changes in periodicity. The stimuli in question are high-pass-filtered click trains where the interval between successive clicks varies. Previously we showed that the cascade autocorrelation model with fixed integration times [13] predicted the pitch percept elicited by a range of click train stimuli, which had proved problematic for conventional autocorrelation models [49], [50], [60]–[63]. Here, we test whether the current model (which generalizes the model reported in [13] by including variable integration times) retains this ability. This is an important question, because a rapid reset of pitch information is apparently in contradiction with the long-term integration used in [13], as was illustrated in the Methods section (Figure 3).

**Figure 8:**
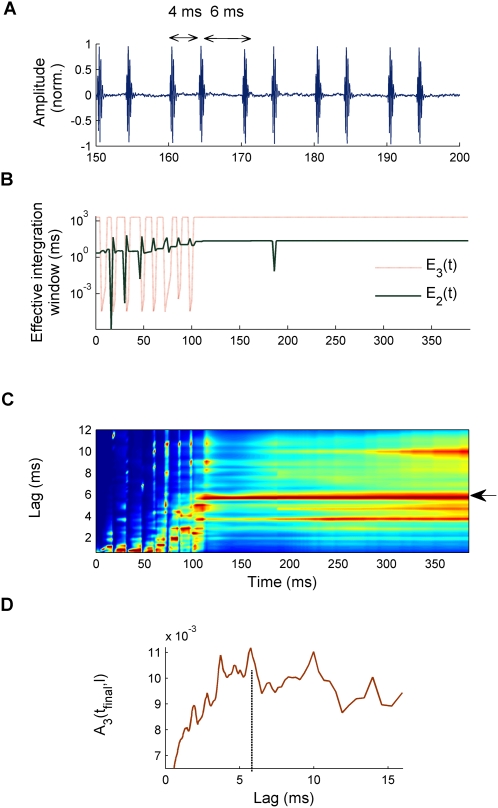
Model response to a high-pass-filtered click train with alternating inter-click intervals [49],[50]. (A) Central portion of the stimulus waveform (the total duration is 400 ms) for a click train with inter-click intervals alternating between 4 and 6 ms after high-pass filtering and the addition of a pink noise background. (B) Effective integration time, *E_2_*(*t*) (green solid line) and *E_3_*(*t*) (red dotted line) at the second and third stages of the model. (C) Model response at the third stage, *A_3_*(*t,l*), normalized and displayed as in previous figures. The arrow marks the lag corresponding to the pitch reported by listeners. (D) Final snapshot of *A_3_*(*t,l*) at *t_final_*. The vertical dashed line corresponds to the average pitch reported by listeners.

As an example, Figure 8 shows the response of the model to one of these stimuli. In this case, the inter-click intervals alternate between 4 and 6 ms, but listeners usually report a single pitch somewhere in between these extremes and closer to the longer interval. Carlyon et al. [49],[50] presented the click trains with a duration of 400 ms. Stimuli were band-pass-filtered with cut-off frequencies of 3900 and 5300 Hz in order to avoid the harmonic spectral components being resolved by the cochlear filters. They also added a pink noise to avoid audible distortion products. Carlyon et al. [50] demonstrated that the combined auditory nerve responses, measured as compound action potentials (CAPs), were stronger for the largest inter-click interval (6 ms) than for the shorter interval (4 ms). Therefore, they suggested that a population of more central neurons, which respond only when their inputs exceed a fixed threshold value, would respond preferentially to the longer intervals, thereby explaining listeners' preference for matching a pitch close to 6 ms.

Figure 8C shows that the predicted pitch of the model (red highlight) varies almost randomly for approximately 80 ms and then progressively stabilizes at a lag in the region of 5.5–6 ms (see horizontal arrow in Figure 8C). Thus, the model prediction is in good agreement with the geometric average of the reported pitch values (shown by vertical dashed line in Figure 8D). While the final snapshot of *A_3_*(*t_final_,l*) (Figure 8D) peaks close to the geometric mean of the reported pitches (vertical dashed line), there are other prominent peaks in *A_3_*(*t_final_,l*) close to this maximum; this is consistent with the large variability in reported pitches for these alternating click trains. A prediction of the model yet to be tested is that no reliable pitch estimate would be possible for stimuli shorter than 100 ms. To conclude, it is worth remarking that this model can similarly account for the pitches of the other click train stimuli considered in [13].

### Cortical Latency of the Pitch Onset Response

**Figure 9:**
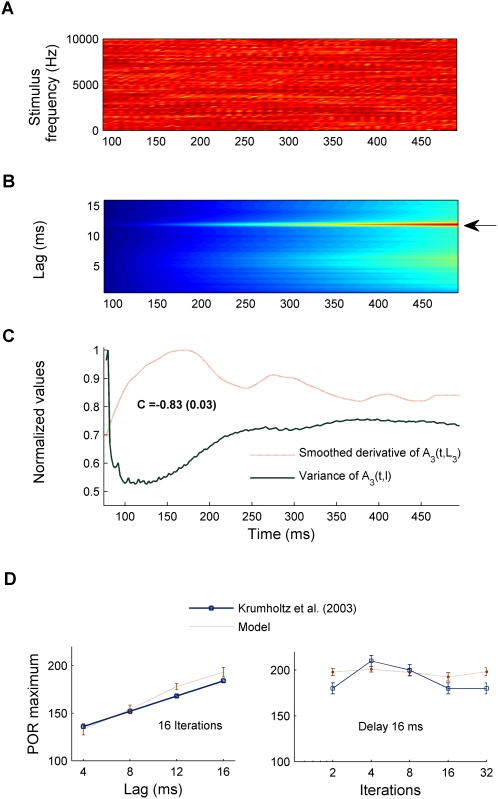
Model evaluation of the Pitch Onset Response (POR). (A) Spectrogram of the final portion of the stimulus waveform; consisting of 500 ms of iterated rippled noise (delay 12 ms, 16 iterations); preceded by uncorrelated noise (not shown). (B) *A_3_*(*t,l*) (without any normalization); colours show activation strength as a percentage of the maximum response. The horizontal arrow indicates the delay corresponding to the reported pitch of this stimulus. (C) Smoothed derivative of *A_3_*(*t,l*); obtained by convolving the model output with the first derivative of a Gaussian function of 60 ms width and 6 ms of standard deviation (dotted red line). Solid green line shows the variance of *A_3_*(*t,l*). *C* is the Pearson correlation coefficient between the smoothed derivative and the variance. (D) Comparison between the model and neuromagnetic results. The solid blue line illustrates the latency of the experimentally measured POR. The dotted red line shows the time at which the maximum of the smoothed derivative is first achieved (within a 2% of tolerance in this value). The left panel shows the POR latencies as a function of delay when the number of iterations is fixed (16). The right panel shows POR latencies when the delay is fixed to 16 ms and the number of iterations varies.

The model proposed here is not a formal model of neural populations; nevertheless, it is neurophysiologically based (see Methods and Discussion sections). This raises the question as to whether the model can explain aspects of the responses of neural ensembles in a pitch perception task. Krumbholz et al. [37] identified a transient neuromagnetic response in Heschl's Gyrus, which they termed the “pitch onset response” (POR). In their experiment, they used iterated rippled noise (IRN) stimuli with delays of 4, 8, 12 and 16 ms. IRN differs from the RN stimulus described previously in that the delay-and-add process is iterated *N* times. Increasing the number of iterations, *N*, increases the degree of serial correlation and therefore the pitch strength. Figure 9A shows the spectrogram of an IRN stimulus with a 12 ms delay and 16 iterations. Neuromagnetic responses were recorded to the onset of an IRN, which was directly preceded by an uncorrelated noise with the same energy and spectral composition. Recordings showed that the transition from noise to IRN produced a reliable POR with a mean latency of approximately four times the delay, *d*, plus a constant offset of about 120 ms (left panel in Figure 9D, solid blue line). The authors concluded that the POR reflects pitch-related processing within Heschl's Gyrus in the human auditory cortex. This has been supported by other more recent studies [36].

Figure 9B shows the output of the model, (*A_3_*(*t,l*) without any normalization, in contrast to previous plots), for the example shown in Figure 9A. After some time the maximum of *A_3_*(*t,l*) (red colour) stabilises and becomes prominent. The predicted pitch is the reciprocal of *L_3_* = 12 ms, which corresponds to the delay of the IRN stimulus. However, the maximum value of *A_3_*(*t,L_3_*) in Figure 9B emerges gradually. Therefore, there seems to be no obvious correlate of the latency at around 150 ms of the measured cortical response in the model.

A number of previous studies have suggested that the temporal derivative of the neural population responses at lower levels of processing might correlate with the measured activity in higher (i.e., cortical) levels [44],[45],[64]. Therefore we investigated whether the latency of the pitch onset response might correspond to the latency of the peak in the derivative of *A_3_*(*t,L_3_*).

Here, we calculated a smoothed version of the temporal derivative of *A_3_*(*t,L_3_*) by convolving *A_3_*(*t,L_3_*) with the first differential of a Gaussian function (representing connection efficacies to higher areas [44],[45]). We then used the first maximum of this smoothed derivative to predict the latencies of the POR for different pitch values. Figure 9C illustrates the smoothed derivative of *A_3_*(*t,L_3_*) for the example shown in Figure 9A (red dotted line). The derivative has a maximum at approximately 168 ms, which is consistent with the POR latency for this condition. The green solid line shows the variance of *A_3_*(*t,l*) (calculated at each fixed *t*) for the same stimulus. It appears that the variance of *A_3_*(*t,l*), which might be taken to represent the uncertainty of the pitch estimate, reaches a minimum at a similar time as the derivative of *A_3_*(*t,L_3_*) reaches a maximum (in general, however, the smoothed derivative is a more accurate predictor of the experimental latencies). The red dotted line in Figure 9D (left plot) shows the time at which the smoothed derivative of *A_3_*(*t,L_3_*) reaches its first maximum as a function of pitch value, which appears to correlate remarkably well with the POR latencies (solid line).

Krumbholz et al. [37] also found that the POR latency mainly depended on the delay of the IRN stimulus and was influenced little by the number of iterations. The right panel in Figure 9D shows the latencies when the delay is fixed at 16 ms and the number of iterations varies (solid line). Consistent with experimental results, the number of iterations of the stimulus do not significantly affect the smoothed derivative of *A_3_*(*t,L_3_*(*t*)) (dotted line).

**Figure 10:**
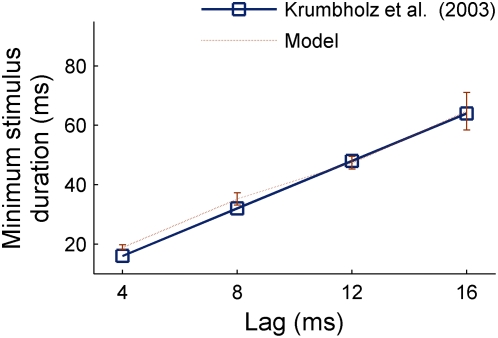
Minimum stimulus duration required to perceive a stable pitch sensation. The solid blue line shows the perceptual results averaged over listeners; and the dotted red line, the mean model predictions.

To conclude, it is worth mentioning that the model also accounts for the minimum duration of IRN stimuli for reliable perceptual discrimination of the pitch, also reported in [37]. The solid line in Figure 10 indicates the average perceptual results. The dashed line shows the duration of the transient period in *A_3_*(*t,L_3_*), i.e., the time window during which the pitch prediction is not stable (e.g. around 100 ms in the stimulus shown in Figure 8C). Clearly, the model simulations match the data extremely well (dashed line in Figure 10). Therefore, the initial period in which the model output varies rapidly seems to correlate with unstable pitch perception. This model prediction might be valid not only for IRN stimuli but also for other pitched sounds.

## Discussion

We propose a neurocomputational model to explain the observed paradox between temporal integration and temporal resolution in the auditory processing of pitch information. Our goal was to capture essential elements in the temporal dynamics of pitch perception within a unified framework. This model is an extension of the autocorrelation theory of pitch perception formulated in terms of equations describing the activity of neural ensembles [51],[65], and extended to include feedback processing.

The principal novelty of the model is the suggestion that top-down connections to sub-cortical areas determine the temporal dynamics of auditory perception, and that this influence is mediated through feedback modulation of recurrent inhibitory circuits. As a result, the responses at each stage adapt to recent and relevant changes in the input stimulus; i.e., feedback in the model essentially determines the dynamics of the “effective” integration window used at each stage. This approach is consistent with the available neuroimaging data: a sustained pitch response (SPR) in lateral Heschl's Gyrus has been shown to adapt to the recent temporal context of a pitch sequence, enhancing the response to rare and brief events [36]. The successful explanation of the latency of the Pitch Onset Response (Figure 9D, left plot) further supports the neurobiological validity of this model. Therefore, we hypothesize that the model captures some fundamental processing aspects of pitch processing, occurring up to Heschl's Gyrus [37]. Consistent with this, a recent study also suggests that the auditory sensory thalamus processes fast changes in speech, which appears to be modulated by slower contextual states [66].

It should be noted that efferent connections to the auditory peripheral model have not yet been implemented, although there is evidence for those connections too [67]. The addition of this connection could provide a method for controlling the cochlear model, a current focus of our investigations.

Although highly idealized, the model uses a minimal set of biologically plausible parameters. The values shown in Table 1 were optimized for generating the correct temporal dynamics of the effective integration windows in the global pitch of non-simultaneous tones, and the pitches of click trains, described in the Results section. Neither the gap detection threshold nor the POR latency experiments were used for parameter optimization; they therefore stand as tests of the generalization of the model.

The current model might thus serve as a basis for more realistic neurophysiological models in the future. In fact, the model responses during the offsets of tones are similar to responses of neurons to amplitude modulated pure tones measured in the superior paraolivary nucleus (SPON) of rats [68]. Remarkably, a short gap between tones was found to produce a significant burst of spikes; i.e., a change in neural activity of several orders of magnitude in less than a millisecond during discontinuities between the tones. Consistent with this data, the model responses *A_n_*(*t,l*) vary very quickly at tone offsets, because the effective integration windows become very short at these discontinuities (Figure 2C). Interestingly, this very fast offset response in SPON neurons is not a feed-forward process, but is modulated by feedback from neurons in the medial nucleus of the trapezoid body, which inhibit the SPON [68]. The model architecture shown in Figure 1 is similar to this type of feedback inhibitory circuit.

In some ways (see Text S1) the model can be understood as a special case of a more general class of models: the *hierarchical generative models* (HGMs) of sensory processing [38],[39],[69]. In the HGM approach, it is assumed that higher areas have access to more abstract and contextualized information, and therefore produce a more refined expectation of the next sensory input. Lower areas deal with more detailed information and generate intermediate predictions [38]. A mismatch between these two predictions generates an error, which propagates from the upper level to the level immediately below and minimizes the *free energy* of the model [38],[69]. This is shown in Text S1, where a comparison between the proposed model and HGMs is presented. Very recently, Kiebel and colleagues also showed that the minimisation of the free energy can be used to invert temporal hierarchies in the processing of bird songs [70].

In summary, we propose a unified model to explain the stimulus-dependency of the time constants of temporal processing in auditory perception. We suggest that one possible role for efferent connections in the auditory system is to detect perceptually relevant changes in the temporal patterns of afferent activity and to adapt the effective processing time constants to the stimulus characteristics. Currently, we are not aware of any studies that have explicitly tested the role of efferent signals in pitch perception, thus, this hypothesis has yet to be tested. Nevertheless, a prediction of the model is that blocking the feedback circuits would impair the ability to separate sounds over time. Recent experimental studies in cortical cooling [71] may provide a methodology for further testing this proposal.

## Supporting Information

Text S1 This section explores the similarities between this study, which does not use a Bayesian inference approach, and the Hierarchical Generative Models (HGMs) of sensory processing.
(0.10 MB DOC)
