# Supplementary material for: Understanding Pitch Perception as a Hierarchical Process with Top-Down Modulation
Source: PLoS Comput Biol. 2009 Mar 6;5(3):e1000301. doi: 10.1371/journal.pcbi.1000301 (PMC2639722; doi:10.1371/journal.pcbi.1000301)
Supplement: Text S1 — This section explores the similarities between this study, which does not use a Bayesian inference approach, and the Hierarchical Generative Models (HGMs) of sensory processing. (0.10 MB DOC) [file pcbi.1000301.s001.doc]

Text S1. Analogy with Hierarchical Generative Models

This section explores the similarities between this study, which does not use a Bayesian inference approach, and the Hierarchical Generative Models (HGMs) of sensory processing, which do. The responses of our model are not probability distributions, because *A_n_*(*t,l*) is not conveniently normalized at each time step. However, in this appendix, we consider the model responses as approximately represented by probability distributions, for illustrating the comparison with HGMs. The next table indicates the equivalence between the symbols used in [38] and here.

**Table S1**. Comparison between the nomenclature in [38] (section 3.7) and of the proposed model.

| **Friston (2003)** | **Model** |
| --- | --- |
| ν*_1_*, ν*_2_*, ν*_3_* ≡ ν Model inputs (ν*_1_*), intermediate causes (ν*_2_*) and top-level causes (ν*_3_*) | Lag *l* at the three model stages |
| θ*_1_*, θ*_2_*, θ*_3_*. Parameters at each level | θ*_1_→* Peripheral model parameters [31, 32]  θ*_2_*, θ*_3_ →* θ*_n_*, τ*_n_*, η*_n_*, μ*_n_*; *n=2, 3*. |
| *p*(ν*_1_*|ν*_2_*). Conditional probability distribution of predicted inputs given the level 2 prediction | Second level predictions of the aggregated auditory nerve activity *A_1_*(*t,l*) |
| *p*(ν*_2_*|ν*_3_*). Conditional probability distribution of level 2 responses given the level 3 prediction | Third level predictions of the second stage response *A_2_*(*t,l*) |
| *p*(ν*_3_*). Probability distribution of level 3 responses. | *A_3_*(*t,l*) |
| μ*_1_=G_1_*(ν_2_*;* θ*_1_*)  μ*_2_=G_2_*(ν_3_*;* θ_2_)  μ*_3_=G_3_*(ν_3_*;* θ_3_)  Mode of the probability distributions in different levels. | *L_1_=l/Max*(*A_1_*(*t,l*))  *L_2_=l/Max*(*A_2_*(*t,l*))  *L_3_=l/Max*(*A_3_*(*t,l*)) |
| Σ_1_, Σ_2_, Σ_3_. Covariance matrix of the probability distributions. | Variances in the responses across the lag axis. |

For further exploring the links with HGMs, it is assumed that *A_3_*(*t,l*) is normally distributed at each time step with respect to the lags variable. For a purely harmonic complex the model responses are symmetric and strongly peaked around the lag of the fundamental; therefore, this simplification is reasonable (Figure S1).


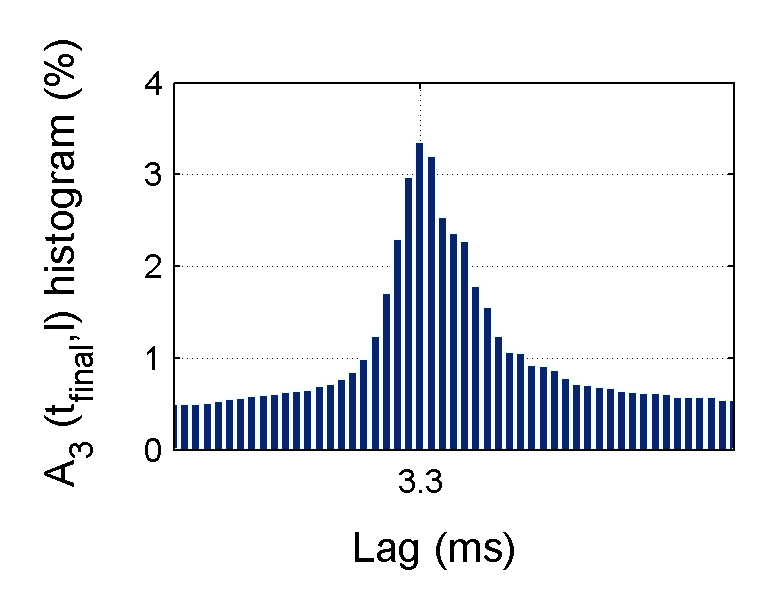


**Figure S1.** Histogram of *A_3_*(*t,l*) for a 3.3 ms pure tone (vertical dashed line); which corresponds to the final model response to the sequence of tones shown in Figure 2.

The proposed model has been presented from the perspective of the recognition, or bottom-up process. However, in HGMs, the generative (top-down) model forms the basis of the formulation and the recognition model is obtained by the *inversion* of the generative model [38], which is performed by the feedback processes. The model presented in this study is straightforwardly invertible, due to the use of a multiplicative gain instead of a sigmoid activation function in equation 3, as is illustrated in this supplementary section. Therefore, a top-down model can be approximately developed [38]. Using Table S1, *A_3_*(*t,l*) can be re-written as the final probability distribution, *p:*

$p\left(\nu_{3}\right)=\frac{1}{\sqrt{2\pi\Sigma}}e^{-\frac{\left(\nu-\mu_{3}\right)^{2}}{2\Sigma}};$ $\begin{matrix} \nu_{3}=\mu_{3}+\varepsilon; & \Sigma=Cov(\varepsilon\end{matrix});$ **(S0)**

where for simplicity the variables ε will be generated by normal distributions with the same variances *Σ*, which are held constant in the model. A single symbol ν represents the lag parameter, which is the same for all three stages; but sub-indices will be preserved for notational convenience (Table S1). Note also that μ*_3_* is the mode of the distribution, not the symbol used in our model (see equivalences in Table S1). Equation 3 at n=3 (omitting the normalisation factor in equation 2 for simplicity, i.e. *g_3_*(*t*)=1+ *_2_/*λ*_2_* ≈1) can be re-written, in the notation of [38], as

**(S1)**

*p*(ν*_2_*|ν*_3_*) can be expressed as a normal distribution, in which the mean is given, at each time step, by a non-linear function of the upper level causes,

where we have defined

$$G_{2}(\nu_{3};\theta_{2})=\left[\nu\mp\sqrt{(\nu-\mu_{3})^{2}-2\Sigma\cdot\ln\left(\varphi_{3}(\nu;\theta_{2})\right)}\right]^{+};$$

.

Note that *_2_* represents here a set of parameters, not only a threshold (see equivalences in Table S1). Therefore, like equation 24 in [38],

$\begin{matrix} \nu_{2}=G_{2}(\nu_{3};\theta_{2})+\varepsilon; & \Sigma=Cov(\varepsilon\end{matrix});$ **(S2)**

Similarly,

$\begin{matrix} \nu_{1}=G_{1}(\nu_{2};\theta_{1})+\varepsilon; & \Sigma=Cov(\varepsilon\end{matrix});$ **(S3)**

where we have defined

$$G_{1}(\nu_{2};\theta_{1})=\left[\nu\mp\right.\left.\sqrt{(\nu-G_{2}(\nu_{3};\theta_{2}))^{2}-2\Sigma\cdot\ln\left(\varphi_{2}(\nu;\theta_{1})\right)}\right]^{+};$$

The model processing can be informally understood as a sub-optimal optimisation method, which can be compared with the standard Expectation-Maximization (E-M) algorithm. In our model, feedforward processes attempt to recognize the likely *cause* (pitch attribute) of the input sound (Expectation), while feedback processes produce an adaptation to recent temporal changes in the input (Maximization). Therefore, like the E-M, this algorithm minimizes an approximate *free energy* [38, 69] after each time step. However, it is worth noting that, in contrast to HGMs [38], the current model does not implement lateral interactions and therefore the precision in the predictions (related to *Σ*^-1^) is not parameterized. The variance of the model response *A_3_*(*t,l*) is related to the latencies of the MEG responses to pitch onset in the Results section.

Figure S2 illustrates the temporal dynamics of this approximation in our model and in the autocorrelation model shown in [13], for an arbitrary sequence of pure tones (Figure 2A). In a *deterministic* recognition situation, the free energy is [38]

$$-F(t)=-\left\langle l(\nu_{1})\right\rangle_{v_{1}}=-\left\langle\ln\left(p(\nu_{1}|\nu_{2})\right)+\ln\left(p(\nu_{2}|\nu_{3})\right)+\ln\left(p(\nu_{3})\right)\right\rangle_{\nu_{1}};$$

where *l*(ν*_1_*) is defined in [38]. Figure S2 shows that the model in [13] (purely feed forward) is inadequate after the offset of the first tone in the sequence, at 100 ms (dashed line). In contrast, the model described here minimises the approximate free energy over time (solid line), as expected.


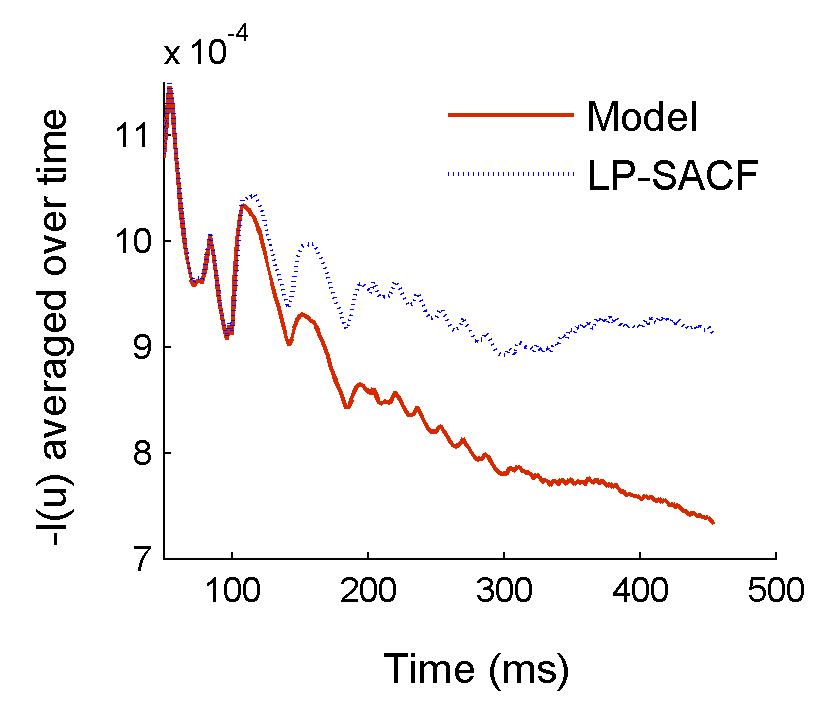


**Figure S2.** Approximate free energy over time (<*-l*(*u*)*>*) when the input stimulus is the sequence of tones shown in Figure 2; for the model of this work (solid red) and for the cascade autocorrelation model in [13] (LP-SACF, dashed line). Deterministic recognition is assumed.
